# Driving with Parkinson's Disease: Exploring Lived Experience

**DOI:** 10.1155/2019/3169679

**Published:** 2019-02-03

**Authors:** Jeffrey D. Holmes, Liliana Alvarez, Andrew M. Johnson, Amy E. Robinson, Kaylie Gilhuly, Emily Horst, Aaron Kowalchuk, Kayleigh Rathwell, Yanni Reklitis, Nolan Wheildon

**Affiliations:** ^1^School of Occupational Therapy, The University of Western Ontario, London, Canada; ^2^School of Health Studies, The University of Western Ontario, London, Canada; ^3^Health and Rehabilitation Sciences, The University of Western Ontario, London, Canada

## Abstract

A growing body of literature has explored the impact of Parkinson's disease (PD) on fitness to drive. As such, evidence now supports the use of specific clinical tests for screening purposes, the predictive validity of risk impressions, and the critical driving errors that predict on-road pass/fail outcomes in this population. However, little is known about the lived experiences of persons with PD as they navigate driving-related concerns such as driving impairments, cessation, potential threats to independence, and community mobility. This qualitative secondary data analysis aimed to explore the driving-related lived experiences of persons with PD. We utilized summative content analysis to identify themes related to driving from transcribed interviews with nineteen community-dwelling individuals with PD who participated in the primary study. Five themes emerged within the analysis: (1) the meaning and significance of driving; (2) driving cessation; (3) modified driving behaviors; (4) factors affecting driving; and (5) accessibility. Participants identified driving as an activity that holds significant importance—both directly (i.e., as a primary activity) and as a means for enabling other activities. This study lays the foundation for the development of client-centred and evidence-informed driving interventions for individuals with PD, as well as the development of driving retirement programs.

## 1. Introduction

In North American societies, driving constitutes the primary means for community mobility and is thus inextricably linked to independence, autonomy, and quality of life [1]. Given the motor and nonmotor symptoms of Parkinson's Disease (PD), individuals with PD are at higher risk for impaired fitness to drive [2], the ability to control a motor vehicle smoothly and cautiously while keeping up with the flow of traffic [3]. In fact, drivers with PD make more critical driving errors than healthy controls—including red light violations and difficulty maintaining lane position [4, 5]—and they are more likely to fail an on-road assessment than age-matched healthy individuals (41% vs 9%) [6]. This growing body of evidence lays the foundation for the development of targeted intervention strategies that can improve the fitness to drive of drivers with PD, as well as develop driving retirement plans that can integrate alternative transportation strategies. However, such intervention strategies must account for the diversity of factors that influence a person's decision to continue to drive or to retire from driving, as well as the diversity of experiences that individuals with PD navigate in their illness trajectory. To date, little is known about the lived experiences of persons with PD regarding their driving, or the prominence driving may or may not have as they navigate life with PD.

In 2011, Rizzo [7] published a case study investigating the meaning ascribed to driving by an individual living with PD, a case study that was among the first published accounts of lived experience and driving in individuals with PD. The case study also included a commentary by the person's treating physician, specifically pertaining to the driving cessation process. A more recent phenomenological study of PD also focused specifically on the experience of driving cessation and included 22 participants with PD and 12 family caregivers living in Australia [8]. Participants reported the experience of anxiety while driving as well as anticipatory anxiety or worry related to driving cessation. These authors stressed the need for active planning on the part of individuals living with PD and their caregivers to address eventual driving cessation [8]. To our knowledge, these are the only two published studies that have investigated the lived experiences of persons with PD as they relate to driving, and both focused on exploring questions around driving cessation. Yet, the extent to which persons with PD perceive driving as a prominent concern resulting from their illness trajectory, or the potentially diverse driving concerns from diagnosis to cessation, remains unexplored.

Given the implications that driving has for independence, autonomy, and social participation, exploring and understanding the lived experiences of people living with PD is critical to the development of targeted interventions and preemptive driving retirement approaches. Therefore, the purpose of the current secondary data analysis is to explore the lived experiences of people with PD surrounding their fitness to drive.

## 2. Materials and Methods

### 2.1. Setting and Context

This study is a secondary analysis of qualitative data collected as part of a primary data set designed to gain a deeper understanding of people with PD's stories of life and their experiences of living with PD [9, 10]. Ethics approval was obtained from the Health Sciences Research Ethics Board at the University of Western Ontario (#103356). The parent study was situated within a social constructivist paradigm and used visual narrative inquiry to gather data via photo elicitation to facilitate participant's telling of stories [11–13]. The first author of this manuscript was also the principal investigator of the parent study, and it was during the analytic interpretation that the need for this secondary data analysis of a driving-focused inquiry was identified, given the prominence of the issue.

### 2.2. Primary Data Set

#### 2.2.1. Participant Recruitment

For the parent study, a convenience sample of participants was recruited via posters displayed within local movement disorder clinics in a midsized Canadian city and from information sessions that were delivered at local PD support groups and regional PD conferences. Individuals were eligible for participation if they were living with idiopathic PD and understood English. Participants were excluded if they experienced cognitive or communication difficulties that impacted their ability to participate in an oral interview or group discussions.

#### 2.2.2. Data Collection

Participants received a digital camera and training and were asked to take photographs over a two-to-four week period that would help to explain their experience of living with PD. Following collection of photographs, participants completed an individual semistructured interview lasting 60–90 minutes. Participant discussion surrounding each photograph was initiated by asking them to describe the photograph, their reasons for taking it, and what the photograph meant to them. All interviews were audio-recorded and transcribed verbatim by a professional transcriptionist. For the purposes of publication and maintaining confidentiality, all participants were provided with a pseudonym.

### 2.3. Secondary Data Analysis

The current study involves a secondary data analysis of the parent study transcriptions, conducted using summative content analysis to capture the prominence of driving as an emerging topic, and the themes related to participants' experience of driving [14]. Each transcript was read and coded by two individuals, identifying instances where participants referred to driving experiences. Coding was completed in an inductive and iterative manner. Members of the research team independently coded their assigned transcripts and then discussed their emergent codes as part of a larger group. Once the code list was agreed upon, transcripts were read again and recoded. Codes were then organized into themes and subthemes based on similarities. QSR International's NVivo 11 qualitative data analysis software was used for coding.

## 3. Results

The interview transcripts of all 19 participants (14 men) from the parent study were included in this secondary analysis. Participants were aged 57 to 79 (*M* = 68, SD = 6.3) and had lived with PD for 2 to 21 years (*M* = 7.3, SD *=* 5.3). Of the 19 participants, 18 included driving-related experiences and/or concerns as part of their experience of living with PD. From these transcripts, five thematic categories emerged within the analysis. The primary themes, in order of frequency, are as follows: Meaning and Significance of Driving, Driving Cessation, Modified Driving Behaviours, Factors Affecting Driving, and Accessibility (Table 1). Themes and subthemes are discussed below with supporting quotations and figures shared by participants.

### 3.1. Meaning and Significance of Driving

**Figure 1:**
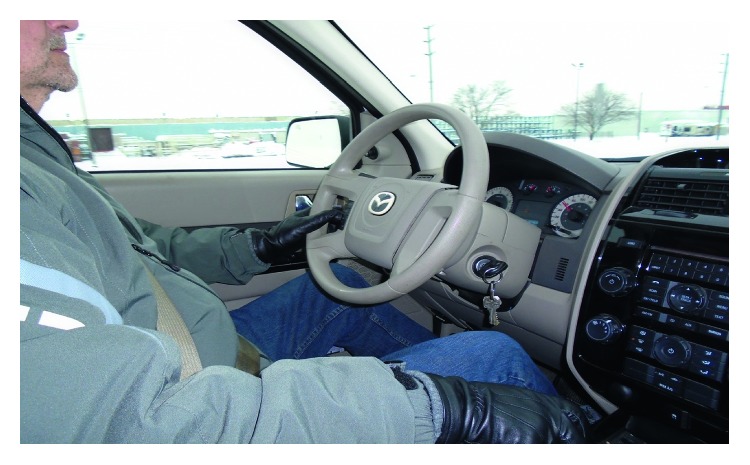
Sandy demonstrated his passion and ability to safely drive while living with PD.

**Figure 2:**
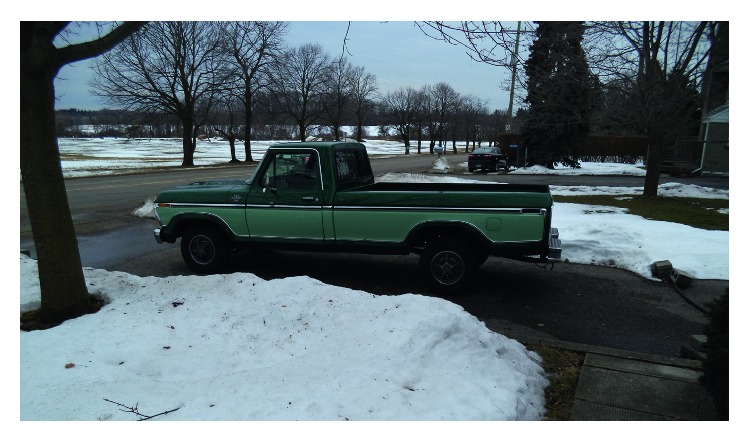
Greg reflected on his truck being a positive thing that brings him joy to look at and drive.

The most frequently discussed theme among participants was their personal conception of the meaning of driving. Thirteen of the participants spoke about the significance of driving in their own lives, and the theme was referenced a total of 56 times. Participants discussed driving as an activity, specifically as one that they enjoyed, but also described driving as a means that enables engagement in other activities such as work or social relationships. For example, Sandy explained “*it could be one in the night, you want to go drive a country road? I'll go. I just love to drive*” (Figure 1). Similarly, Greg spoke about how his truck brings him joy: *“just a feel good old truck. Just a positive thing for me to look at get in and take for a drive”* (Figure 2). On the other hand, Kelly expressed *“I have got to drive to work, I cannot bus because it's way out in the middle of nowhere.”* Similarly, Corey explained *“I do not drive for pleasure much anymore. If I drive, it's because I've got to go somewhere.”*

Furthermore, participants spoke about how their ability to drive was strongly tied to their identity and how they believed others perceived them. Sandy expressed these feelings when describing how he has not told people at work about his PD by stating *“They would question me I think, am I competent to do the job…I do not want people to start questioning and say, oh… maybe you can't drive....is it safe for us to have you in here?”* Similarly, some participants discussed the importance of driving in relation to their feelings of independence and freedom. Rebecca explained *“my car is my independence to go every place I go, exercise classes, visiting with friends…losing that I would have trouble asking for friends to pick me up twice a week to be in exercise class and twice a week to Tai-chi and another time for women's group. Maybe, it will come to that at some point but I'd like to be able to drive as long as I can.”*

### 3.2. Driving Cessation

Thirteen participants discussed driving cessation, which was referenced a total of 46 times. Participants described the impact that driving cessation had on themselves; on others such as family and friends; and finally they reflected on their readiness to give up their license. Sandy explained, *“if [my license] is taken away it will be very difficult to deal with. If I can't skate when I go out, no big deal. I try, okay. But this here, when [my license] is taken away, that will be a big deal, if it's taken away.”* Similarly, participants discussed the impact driving cessation has had on their family members and friends. For instance, Sam explained *“it's not fair to (spouses name), she does all the driving, no discussion… it's an awful load on her shoulders”* highlighting a shift of driving responsibility from Sam to his wife. Similarly, Corey noted, *“I used to enjoy driving the car…. I would have driven 70 or 80 percent of the time, now it's the other way around (i.e. spouse now does the majority of driving although she does not particularly enjoy doing so).”* While some participants had already experienced driving cessation, others discussed the situation in prospective terms. Alex expressed concerns regarding his future *“my wife does not drive she never got her license… so, we're saying, okay, what's going to happen 5 years out because I'm so active? I still have to be out and about. We have not crossed that bridge yet. She might get her license.”* Similarly Sandy noted, “…*in the future, I do not know how I'm going to react to being housebound or being bound to a bus or a taxi, or depending on my daughter or my son to come get me. Telling me, oh, I can't make it for an hour.”* Some participants also discussed their readiness and willingness to cease driving, such as a feeling of relief if the decision was made for them. For example, when asked if he was *“feeling comfortable behind the wheel,”* Sam responded *“No. So when they came and said we're taking (your license) away it was actually a relief.”*

### 3.3. Modified Driving Behaviors

Twelve participants discussed how they have adapted their driving behaviours in order to continue driving. A total of 52 references were made to this theme as participants described how they avoided various driving conditions or implemented compensatory behaviors. Specifically, participants discussed how they tend to avoid certain environments such as big downtown cores with a lot of traffic. Sandy expressed *“if there's a lot of traffic, I'll just stick into the one lane and go where I have to go. I don't like changing lanes.”* Several participants also discussed their aversion to driving long distances, explaining the need for frequent breaks. Rebecca explained, *“I got into a construction zone. It took me an extra hour and a half. By the time I got to my son's place I was shaking. It was too long in the car.”* Similarly, Alex explained, *“more than two hours, I have to pull off and stretch and get out for a walk.”* As well, some participants stated that they no longer drive in the dark, or in winter weather. In addition to the several situations that participants tended to avoid, they also discussed compensatory strategies that they had taken up in order to maintain their ability to drive. Some examples of this include planning out route rest stops and limiting turning left in heavy traffic. For instance, Alex explained *“I kind of plan out my trips. If I'm driving to (city), okay, I'll stop here, here, and here….and if you run into traffic problems before that, then you're going to alter that…but I need to stop every couple of hours” Moreover,* Sandy explained *“If there's a whole pile of cars coming straight here and I'm waiting to turn left that bothers me. I prefer not to have to turn left against the traffic anymore…I try to make sure I get the flashing (advanced turn signal) or I will not go that way.”*

### 3.4. Factors Affecting Driving

Eleven participants spoke about various personal factors that impacted their driving ability, including PD-related symptoms, the impact of medications, the influence of fatigue, and their experiences of anxiety while driving. For six participants, the most concerning factors were symptoms directly related to PD. Julian described freezing in his hands, a common symptom associated with PD [15], when stating, *“I was driving … and both my hands locked…. I was just before the (major highway) coming up to (busy road), and my thought as I was hitting the overpass is, I wonder what would happen? And the other one locked. And both my hands locked and I had to put my hands on the wheel and drive home like that in pain.”* Similarly, Corey discussed how reduced concentration, another common symptom of PD [15], affected his driving, *“I have to really force myself to stay concentrated when I'm driving … with both hands on the wheel and pay attention to what your doing.”* Several participants spoke about how their medication and the timing of it can have effects on their ability to drive. For example, Alex explained that he alters the timing of his medication to ensure he is “on” while driving, he stated “*you may alter your daily exposure to (medication)…if I'm going to be in stressful situations make sure your done the meds at least an hour before (driving).”* In contrast, Rebecca commented on her concerns about the effects of medication on her alertness while driving, saying “*(I do not) want to take (medication) before I drive because I'll be drowsy.”* Brian also explained that “*one of the medications can make you fall asleep when you're driving”* and recalled a particular case where he had fallen asleep at the wheel, drifted to the other side of the road, and woke up to a car approaching head on. *“I opened my eyes and he was right in front, and I was in his lane. Then all of a sudden we were over to the other side. I was going fairly fast, too, I guess...if it had not been for (passenger) pulling on the wheel and getting (the car) back on the right side of the road, there would have been a dreadful accident.”* Others spoke about how fatigue can interfere with their driving ability. Corey commented that his wife *“drives the car a lot of the time now because I get too tired,”* and Rebecca explained that *“We've been going down there (city in the USA) for 10 years and spending two to three months away in the winter. And we're not going this year because the drive is just too much. Driving is tiring and (spouse) can't do it and I can't do it so it's a big change for us.”* Finally, several participants discussed how certain driving situations can increase anxiety and in turn may negatively impact driving by exacerbating PD symptoms. For example, Julian stated *“in the last couple of years there have been times when I've been very anxious, very anxious about things, and when I'm driving, sometimes in a new area or something, I am very anxious.”* Similarly, Alex explained, *“When my stress level gets up because of traffic or whatever, I find the tremors really get worse. I used to be able if I really concentrated, I could control it. It's getting to the point now where I'm not able to control it as much so I try to stay out of stress situations. Especially driving, that's bad.”*

### 3.5. Accessibility

**Figure 3:**
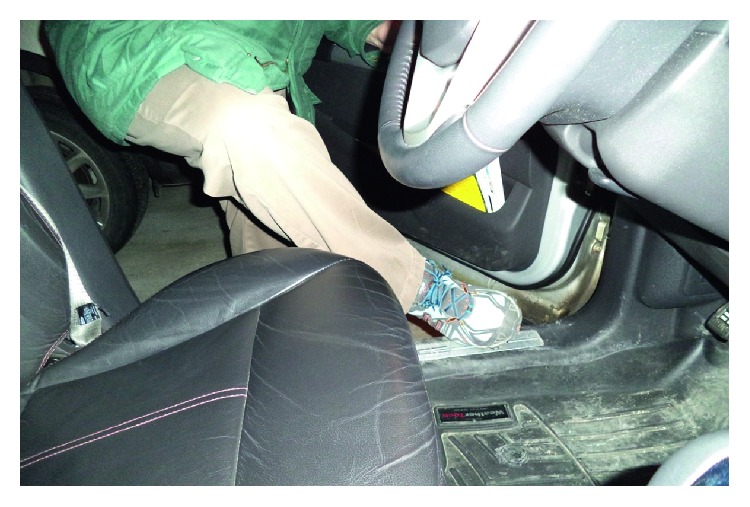
Drew described how he is the slowest person to transfer into and out of a vehicle and that he finds it difficult to lift his foot high enough.

**Figure 4:**
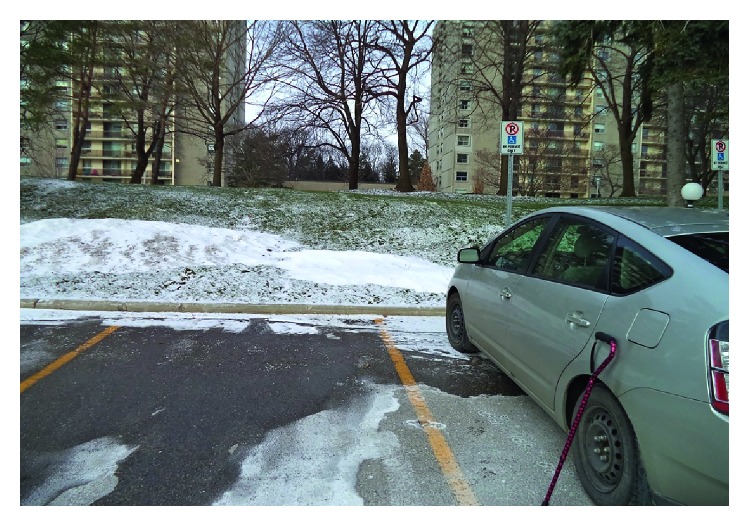
Jessie highlighted how accessible parking enables her engagement in instrumental activities.

Nine participants discussed the final theme of accessibility, specifically in reference to accessibility of the vehicle and their experiences with community mobility. Some participants discussed challenges they had experienced when getting in and out of their vehicles; for example, in discussing the photo presented in Figure 3, Drew noted *“I'm always the slowest one getting in and out of the car…that lift over height, I kind of catch my foot on it…I never seem to lift (foot) high enough to really clear it. I'm always hitting it.”* Drew also noted difficulties with donning his seat belt “*I find it hard to pull it until I get it over this way. This range to this range is kind of hard because I'm pulling with this hand that has a hold of the front of the buckle. I'm trying to get this hand through the seatbelt at the same time as I push it. I've got to get the belt all the way extended…I'm the slowest one to get the seatbelt done up…. They're all waiting for me. They're all clicked in…”* Others discussed the importance of accessible parking and how access to such parking helped to enable instrumental activities such as grocery shopping (Figure 4); for example, Jessie expressed the following: *“Sometimes I drive over to the mall. The disabled spots are right by the door…usually I get one, or I drive around until I do, or I go home…it's disabled parking… that's really a real freedom.”*

## 4. Discussion

The findings of this secondary data analysis highlight the prominence and significance of driving as in the lived experience of persons with PD. When asked to capture and reflect on their lived experiences, nearly all participants in the parent study specifically addressed driving and elaborated on the implications that impaired fitness to drive has for them. This secondary data analysis illuminate the relevance of driving for the participant's independence, sense of identity and competency, as well as the factors that cause concern or increase anxiety.

The meaning of driving, as described by study participants, was found to be broad and diverse. Participants described driving as an enjoyable activity and/or one that enables their independence and community mobility. As such, being able to drive was described as important for the participants' identity (e.g., being perceived as competent) and independence. It is important to note that, although previous research has identified driving as an instrumental activity of daily living, individual views of the same activity may vary substantially from one person to the next, which is consistent with our findings [16]. However, the findings of this study illustrate the relationship between driving and independence, as well as the potential stigma and negative perceptions that participants may experience as a result of losing their license. This finding aligns with those of Fonda and colleagues [17], who investigated the impact of driving reduction and cessation on the mental health of older American adults more generally. In their study, Fonda and colleagues [17] found that participants who stopped driving or reduced their driving were at a greater risk of worsening of depressive symptoms. Furthermore, they posit that driving cessation may be associated with a stigma of dependency, much like the one described by participants with PD in this study. Although Fonda et al. [17] explored depressive symptoms in an older adult population more generally, the findings of this study support similar experiences of stigma by those with PD. In addition, participants in our study identified driving as critical for meaningful engagement in activities that are critical to the participants' sense of independence and autonomy. Such perceptions among persons with PD are not surprising given the value that Western societies ascribe to driving as an instrumental activity of daily living that enables individuals to engage in activities they identify as crucial to maintaining their quality of life [18–20]. As such, driving cessation represents a problematic transition with foreseeable negative consequences for their quality of life.

Participants in this study discussed driving cessation with respect to the impact it has on themselves, on others in their life, as well as their readiness to cease driving. Although some participants recognized that driving may become anxiety-provoking to the point that license revocation may come as a relief, several participants described it in terms of a burden for loved ones and a decrease in their independence. These findings speak to the pervasive effect that driving cessation can have within a family or social unit [21] and are reflective of findings in the literature pertaining to older adults in general as it relates to driving cessation and health outcomes. In a systematic literature review of the influence of driving cessation on the health outcomes of drivers 55 years of age and older, Chihuri et al. [22] identified that driving cessation was related with general health decline, declines in physical, social, and cognitive function, increased risks of long-term care admission, and mortality. Interestingly, four participants indicated a readiness for driving cessation, one of whom expressed relief over license revocation as it decreased anxiety over this difficult decision. This finding may provide evidence in support of participants' insight into their deficits and their awareness of driving as safety-critical activity. Of particular relevance is the fact that participants' conflicting perceptions around driving cessation point to a need for viable, accessible, and efficient alternative transportation options that can contribute to maintaining independence and preserving identity.

Previous research has suggested that voluntary driving cessation may be related to the availability of alternative transportation [23] and also suggests that a lack of discussion prior to driving cessation can lead to a sudden (and involuntary) loss of license [24]. Not surprisingly, involuntary loss of license has been shown in previous research to be a traumatic experience, due to an abrupt loss of independence and sense of identity [8, 24]. Thus, the development of adequate alternative transportation solutions would benefit individuals with PD and could facilitate transition from “driving cessation,” perceived by participants as a problematic transition and towards “driving retirement,” a process of pre-emptive and person-centred planning for positive transitions.

Twelve of the 19 participants in this study described modifications they made to their driving behaviors in order to cope with the demands of driving. For example, some spoke of avoiding high traffic areas or avoiding left turns where an advanced signal was absent. As well, some spoke about limiting the amount of time they spend driving as it results in fatigue or exacerbation of symptoms. This provides supporting evidence for previously reported quantitative research that suggests individuals with PD may restrict driving duration [25], driving frequency, and distance [26], as well as driving at night, during bad weather, or in high density traffic situations (e.g., highways or at rush hours) [26–28]. Although many of these accommodations are also reasonable adaptations to the effects of typical aging, the accommodations highlighted in the present research were made in response to the unique symptoms of PD. For example, with regards to driving for long distances (or for long durations), participants spoke about specific symptoms (e.g., tremors) that worsened with sitting in the car for too long or processing speed as affecting left turn maneuvers. In addition to avoidance, the current study brings to light the unique compensatory strategies used by individuals with PD. Specifically, several participants spoke about the impact that planning had on their ability to drive. Participants discussed the importance of planning their medication so that periods of peak effectiveness coincided with when they wanted to drive, planning their route to avoid certain turns and include rest stops, and planning their destination with respect to accessible parking. These findings are significant as they illustrate the unique compensatory strategies used by individuals within this population but also their awareness of potentially critical driving errors that may result from PD-specific impairments. Difficulties with concentration, hands locking on the steering wheel, tremors, and pain were among the factors discussed by participants as problematic, and previously reported to put individuals with PD at a higher risk of driving errors [29].

While driving anxiety has been self-reported by older adults in the general population [30], the current study is only the second qualitative study to report driving anxiety among individuals with PD [8]. There remains, however, some equivocation within the literature as to whether or not driving anxiety in older adults is predictive of on-road safety. This is likely because those who experience anxiety while driving tend to avoid it [30]. This trend was evident in the current study as participants mostly discussed anxiety as a deterrent to driving, rather than anxiety impacting their perceived fitness to drive. These findings suggest the need for a large-scale study of driving anxiety in older adults and those living with PD.

Finally, the most frequently discussed factor affecting participants' perceived ability to drive was their medication—both with regards to medication side-effects and medication timing. Some participants suggested that their medication improves ability to drive as it temporarily relieves symptoms. In contrast, others indicated that they avoid driving after taking their medications due to drowsiness. Although previous research has reported a positive correlation between levodopa dosage and driving errors [4], the different effects of medication reported by participants in the present research suggests that the use of medication as a proxy for disease severity needs further empirical validation.

Finally, participants reflected on vehicle accessibility as a determinant of their driving ability. Specifically, some participants discussed how they had difficulty exiting their vehicles from low seats. Some participants also discussed the importance of accessible parking and how they sometimes experience a shortage of available spaces, limiting their participation in activities like shopping. Although past research has not focused specifically on individuals with PD, previous research has highlighted the impact of design on vehicle accessibility and usability, including getting into and out of the vehicle [31]. The present research also illustrates the importance of community accessibility (e.g., accessible parking spots) as this may enable other activities [32]. Research that outlines individuals' perspectives of accessible parking can serve to inform future policy and design of public spaces.

### 4.1. Limitations

While this study provides valuable information pertaining to the lived experiences of people with PD surrounding their fitness to drive, it is not without limitations. First, the original parent study intended to explore the lived experience of PD more broadly, thus the topic of driving was not specifically probed by the researchers. Although this investigation did elicit abundant information from participants regarding their lived experience as it pertains to driving which warranted further exploration, the richness of data may have been enhanced if the topic of fitness to drive was explicitly investigated, a limitation that can be addressed in follow-up studies building on this research. A second limitation of this study is that the sampling approach that was undertaken resulted in a relatively homogeneous sample, which may not capture a complete variety of perspectives and experiences. For example, 14 of the 19 participants in the original study were men, all of the participants were from a midsized Canadian city, and 17 of 19 participants were either married or living with a partner. As a result, the depth of data may be limited, thus future research should focus on having a more diverse sample, with equal representation of gender, marital status, and geographic areas.

## 5. Conclusions

Driving has been identified as an activity that holds significant importance in the lives of individuals with PD. Despite being an important activity, driving (specifically fitness to drive) can be markedly impaired by PD [29]. The findings of this study illustrate the commonalities as well as the variety of challenges and experiences that individuals in this population encounter with respect to their fitness to drive. Individuals spoke about their personal meaning of driving, driving cessation, modified driving behaviours, factors affecting driving, and accessibility. The findings uncovered in this study provide contextualized meaning to the effects of such impairments and allows health care providers to better understand the lived experience of this topic, providing them with new knowledge to inform various areas of practice, such as the design of targeted interventions, as well as the development of person-centred driving retirement plans, including the timely referral to driving-cessation programs.

## Figures and Tables

**Table 1:** Emergent themes (presented in order of frequency) and subthemes.

Theme/subtheme	Number of participants who discussed theme/subtheme	Total number of references to theme/subtheme
*Meaning and significance of driving*	**13**	**56**
Driving as an activity	8	18
Enabling other activities	8	19
Identity	6	9
Independence	3	10
*Driving cessation*	**13**	**46**
Impact on driver	6	17
Impact on others	9	19
Readiness	4	10
*Modified driving behaviours*	**12**	**52**
Avoidance		
Environments	8	12
Long distances	7	11
Nighttime	2	4
Weather	3	8
Compensatory mechanisms	4	17
*Factors affecting driving*	**11**	**43**
PD-related symptoms	6	12
Medication	7	9
Fatigue	5	12
Anxiety while driving	5	10
*Accessibility*	**9**	**26**
Car accessibility	4	13
Community mobility	5	13

## Data Availability

The qualitative data used to support the findings of this study are included within the article.
